# Preparation and Characterization of Electrospun EVOH/Ti_3_C_2_ Composite Fibers

**DOI:** 10.3390/polym16050630

**Published:** 2024-02-26

**Authors:** Xiang Li, Qiao Xu

**Affiliations:** Key Laboratory of Functional Fibers and Intelligent Textiles, Yuanpei College, Shaoxing University, Shaoxing 312000, China

**Keywords:** EVOH, Ti_3_C_2_, electrospinning, structure, properties

## Abstract

In this work, the EVOH/Ti_3_C_2_ composite fibers were prepared via electrospinning and the effect of added Ti_3_C_2_ on the structure and properties of electrospun EVOH fibrous membranes was further investigated. The morphology, crystal structure, thermal properties, wettability, tensile properties, as well as air permeability and water vapor permeability of as-prepared EVOH/Ti_3_C_2_ composite fibers were studied. The Ti_3_C_2_ is uniformly loaded onto the surface and inside the composite fiber and affects the fiber diameters. Furthermore, The Ti_3_C_2_ self-orients along the fiber axis and does not change the crystal structure of the electrospun EVOH fibers, improving the crystallinity and thermal stability of the electrospun EVOH/Ti_3_C_2_ fibrous membranes. With the increase in the Ti_3_C_2_ concentration in the electrospinning polymer solution, the addition of Ti_3_C_2_ not only rapidly improves the wettability of the fibrous membranes, but also enhances their air permeability, compared with the pristine electrospun EVOH fibrous membranes. The experimental results provide theoretical guidance for the preparation of Ti_3_C_2_ composite fibers, and also expand the application of electrospun EVOH and EVOH/Ti_3_C_2_ fibrous membranes.

## 1. Introduction

Electrospinning is a straightforward and prominent technique for the one-step preparation of nanoscale polymeric fibers, which has been extensively applied in drug delivery [1,2], energy storage [3,4], filtration [5,6], tissue engineering [7,8], separation and absorption [9,10]. In view of the wide range of spinnable materials and convenient modifications available within electrospinning, electrospun composite fibers have enormously expanded the versatility of electrospinning in the fields of textile, aerospace, agriculture, biology, defense and security [11,12] due to their high specific surface area, high porosity and interconnected pores [13,14].

Poly(ethylene-co-vinyl alcohol) (EVOH), as a semi-crystalline random copolymer composed of vinyl alcohol and ethylene blocks, is often employed in food packaging owing to its excellent gas resistance, chemical resistance and high transparency [15,16]. However, some completely different applications are found in electrospun EVOH composite fibers, such as filtration and biomedicine [17,18]. Mondragón et al. [19] prepared electrospun poly(ethylene-co-vinyl alcohol(EVOH)/thermoplastic starch (TPS) blend nanofibers and found that the crosslinked EVOH/TPS fibrous membranes exhibited a superior fluid uptake ability (with 20 wt% of TPS) and superior barrier properties (with 20 and 40 wt% of TPS) for potential dressing materials in comparison to those observed in pristine electrospun EVOH fibrous membranes.

Ding et al. [20] fabricated a novel rechargeable N-halamine antibacterial material by functionalizing electrospun EVOH nanofibers with dimethylol-5,5-dimethylhydantoin (DMDMH). The as-prepared DMDMH functionalized EVOH nanofibers membranes provided rechargeable chlorination capacity, high inactivation efficacy against bacteria, high filtration efficiency under low air resistance, and robust mechanical properties. Lu et al. [21] designed the superelastic and superhydrophobic thermoplastic polymeric nanofibrous aerogels (NFAs) for the removal of pollutants from water. Silane-coated EVOH NFAs exhibited a superior absorption capacity (40–92 g/g) for a variety of organic pollutants, which could be used in substantial industrial water purification applications. Hence, the application development of electrospun EVOH fibers has been actively underway.

Recently, MXenes have attracted a great deal of attention because of their high electrical conductivity, excellent solvent compatibility and stability, electrochemical behavior, hydrophilicity, and mechanical strength, which are compared to clay, graphene and GO [22,23]. The general formula of MXene is M_n+1_X_n_T_x_, where M indicates an early transition metal, X signifies carbon and/or nitrogen and T indicates a surface functional group including O, F, and OH [24]. Among them, Ti_3_C_2_, as the most commonly studied MXene, was first reported in 2011 [25] and later found to have antibacterial and antifouling properties, like silver [26,27]. Some research has attempted to coat Ti_3_C_2_ MXene onto fibers for wearable energy storage applications, but the Ti_3_C_2_ flakes were easily detached from the fibers [28]. It is useful to embed Ti_3_C_2_ into the fibers to form uniform composite fibers. Levitt et al. [29] produced a free-standing Ti_3_C_2_T_x_ MXene/carbon nanofiber electrode by electrospinning Ti_3_C_2_T_x_ MXene flakes with polyacrylonitrile (PAN) and carbonizing the fiber networks. Electrospun MXene/carbon nanofibers could suffer from the active material delaminating from the substrate during folding or bending, unlike coated electrodes, which are stable and durable composite electrodes.

In addition, Schauer et al. [30] produced electrospun poly(acrylic acid) (PAA)/Ti_3_C_2_, polyethylene oxide (PEO)/Ti_3_C_2_, and poly(vinyl alcohol) (PVA)/Ti_3_C_2_ fibers and compared the effects of 1 wt% Ti_3_C_2_ addition on the three polymers, and found that Ti_3_C_2_ affected the solution properties of the polymer, especially the diameter of the fiber. It can be seen that the production of electrospun Ti_3_C_2_T_X_ MXene composite fibers can further broaden the applications of Ti_3_C_2_T_X_ MXene with the aid of the unique characteristics of electrospun fibers. Ti_3_C_2_T_X_ MXene, as a new type of two-dimensional nanomaterial, holds regulable physicochemical properties, good biocompatibility, and outstanding photothermal conversion performance, showing a rapid expansion trend in various application fields such as energy storage, catalysis, electronics, electrochemical, electromagnetics, sensing and biomedical applications [31,32,33]. Moreover, Ti_3_C_2_T_X_ MXene exhibits the antibacterial ability to physically damage bacterial membranes and chemically induce oxidative stress [34], and can be further combined with other materials to obtain multi-functional materials. Thus, in view of the unique properties of EVOH and Ti_3_C_2_T_X_ MXene, the multi-functional fibrous membrane can be prepared by adopting electrospinning technology, with potential use in bioprotective materials, dressing materials, etc.

Herein, the electrospun EVOH/Ti_3_C_2_ composite fibers were first prepared and the effect of added Ti_3_C_2_ on the structure and properties of electrospun EVOH fibrous membranes was investigated. This work focuses on the preparation of Ti_3_C_2_ composite fibrous membranes and characterizations of their structure and conventional properties. The morphologies of electrospun EVOH/Ti_3_C_2_ composite fibers with different Ti_3_C_2_ concentrations in the electrospinning polymer solutions were characterized. Moreover, the crystal structures of the electrospun EVOH/Ti_3_C_2_ composite fibers are presented. The properties of the composite fibrous membranes were evaluated, including thermal property, wettability, tensile properties, air permeability and water vapor permeability.

## 2. Experiments 

### 2.1. Experimental Materials

Poly(ethylene-co-viny alcohol) (EVOH) (F171B) was obtained from Kuraray Co., Ltd., Tokyo, Japan. Isopropyl alcohol (IPA) was purchased from Aladdin Chemistry Co. Ltd., Shanghai, China. The 10 wt% EVOH solution was prepared by dissolving EVOH particles in a mixture of IPA/water with a weight ratio of 7/3 at 75 °C for 4 h. Various amounts of Ti_3_C_2_ dispersion with a known concentration of 25 mg/mL were added to the measured EVOH solution prepared previously. The Ti_3_C_2_ concentrations were selected based on the reported research [29,30]. Uniform EVOH/Ti_3_C_2_ solutions with 2.5 wt%, 5 wt% and 10 wt% Ti_3_C_2_ concentrations were obtained by calculating the proportion of polymer solutions.

### 2.2. Preparation of the EVOH/Ti_3_C_2_ Fibers

The EVOH/Ti_3_C_2_ polymer solution was transferred into the syringes at a constant flow rate of 3 mL/h. A high-voltage power supply with an applied voltage of 15 kV was supplied to the spinneret and collector. The distance between the needle tip to the collector was 15 cm. The experiments were carried out at a relative humidity of 45 ± 5% and at room temperature. During electrospinning, the polymer droplets formed Taylor cones under the action of electric field forces. When the electric field forces acting on the droplets overcame the surface tension and viscoelasticity of the polymer droplets, a jet was formed at the top of the droplet cone. Under the action of an external electric field, the jet was highly stretched axially by the electric field force, forming a short distance stable motion. Then, the charges on the surface of the jet repelled each other, causing the jet to produce lateral displacement and enter an unstable motion stage. Finally, the jet flied towards the collector in a spiral whip trajectory, and formed micro–nano fibers with the continuous volatilization of solvent [35,36]. The prepared EVOH/Ti_3_C_2_ fibrous membranes were dried and used for the characterization of the structure and properties. Here the as-prepared electrospun EVOH/Ti_3_C_2_ fibers with 2.5 wt%, 5 wt%, 10 wt% Ti_3_C_2_ concentrations in the electrospinning polymer solution are simply named EVOH/Ti_3_C_2_-1, EVOH/Ti_3_C_2_-2, EVOH/Ti_3_C_2_-3, respectively.

### 2.3. Characterization

Morphology of the electrospun EVOH/Ti_3_C_2_ fiber was observed using a scanning electron microscope (SEM, S-4800, Hitachi Ltd., Tokyo, Japan) after gold coating. The SEM morphology was taken by randomly selecting four areas of the fibrous membrane. The average fiber diameters were measured from the SEM images of more than 100 fibers by the image visualization software (Image J v1.43) (National Institutes of Health, Bethesda, MD, USA). The error bars for the fiber diameters were based on the standard deviation of the record. The elemental distributions of electrospun EVOH/Ti_3_C_2_ composite fibrous membranes were characterized by energy-dispersive X-ray spectroscopy (EDS, Bruker Quantax 400) (Bruker Instruments, Inc., Billerica, MA, USA). 

The crystal structure was characterized by X-ray diffraction (XRD) and Fourier transform infrared spectroscopy (FTIR). XRD measurements were recorded at the beam line BL16B1 of Shanghai Synchrotron Radiation Facility (SSRF, Shanghai, China). The X-ray wavelength is 0.124 nm and a Mar 165 CCD detector with 2048 × 2048 pixels was used to collect two-dimensional (2D) patterns. The 2D patterns were accumulated over periods of 50 s. Fit 2D software (https://www.esrf.fr/computing/scientific/FIT2D/, accessed on 20 February 2024) was applied to transfer 2D patterns into 1D profiles. FTIR spectra were recorded based on the transmission mode using a Thermo Nicolet 6700 FTIR Spectrometer (Thermo Fisher Scientific Inc., Waltham, MA, USA) in the wavenumber range of 400–4000 cm^−1^ at room temperature. The XRD and FTIR tests were carried out in three randomly selected places of the fiber membrane, respectively.

Thermal properties of electrospun EVOH/Ti_3_C_2_ composite fibrous membranes were measured using a differential scanning calorimeter (DSC4000) (PerkinElmer, Inc., Waltham, MA, USA), whose tests were set to heat up from 30° to 240° at a rate of 10 °C/min and then cool down again at the same rate to 30°.

The wettability of electrospun EVOH/Ti_3_C_2_ composite fibrous membranes were estimated using a contact angle meter (Kruss DSA100) (Kruss GmbH, Hamburg, Germany) with a droplet of 3 μL at room temperature.

Tensile properties of electrospun EVOH/Ti_3_C_2_ composite fibrous membranes were performed using a single fiber tensile testing machine (YG005E) (Wenzhou Fangyuan Instruments Co., Ltd., Wenzhou, China) with a 200 N load cell at room temperature. The membranes used for testing were cut into strips with a length of 30 mm and a width of 5 mm and extended at a constant cross-head speed of 0.1 mm s^−1^ with a gauge length of 20 mm. Each sample was repeated five times to obtain an average value and further ensure the repeatability.

The air permeability of electrospun EVOH/Ti_3_C_2_ composite fibrous membrane was tested based on ASTM D 737 standard [37] using a fabric permeability tester (YG461E) (Ningbo Textile Instrument Factory, Ningbo, China) with a test pressure difference of 100 Pa. The samples were cut into a circular shape with a diameter of 10 cm. 

The water vapor permeability of electrospun EVOH/Ti_3_C_2_ composite fibrous membrane was assessed based on ASTM-E96 [38] desiccant method by employing a water vapor transmission tester (YG(B)216-Ⅱ) (Wenzhou Darong Textile Instrument Co., Ltd., Wenzhou, China). A circular cup containing 33 g of CaCl_2_ was placed in a testing chamber at 38 °C and 90% relative humidity with a wind velocity of 1 m s^−1^. WVTR was calculated based on the following equation [39]:

(1) $$WVTR=\frac{m_{2}-m_{1}}{s}\times 24$$

where WVTR indicates the water vapor transmission per square meter every day (24 h) and is expressed in kg m^−2^ d^−1^, *m*_2_ − *m*_1_ is the weight change of CaCl_2_ in the test assembly in 1 h, *s* is the effective area of specimen. For each sample, WVTR and air permeability values were measured at least five times.

## 3. Results and discussion

### 3.1. Morphologies of Electrospun EVOH/Ti_3_C_2_ Composite Fibers

**Figure 1:**
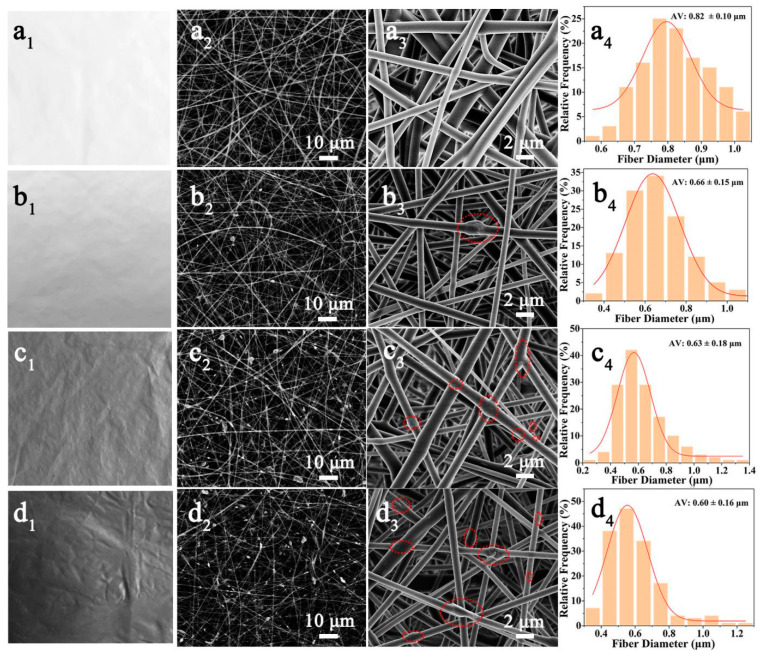
Morphology of electrospun EVOH fiber with increasing concentration of Ti_3_C_2_ in the electrospinning solution: (**a_1_**–**a_4_**) 0 wt%, (**b_1_**–**b_4_**) 2.5 wt%, (**c_1_**–**c_4_**) 5 wt%, (**d_1_**–**d_4_**) 10 wt%. (**a_1_**,**b_1_**,**c_1_**,**d_1_**) Optical images, (**a_2_**,**b_2_**,**c_2_**,**d_2_**) SEM images at low magnification, (**a_3_**,**b_3_**,**c_3_**,**d_3_**) SEM images at high magnification, and (**a_4_**,**b_4_**,**c_4_**,**d_4_**) fiber diameter distributions of electrospun EVOH/Ti_3_C_2_ fibrous membranes.

The Ti_3_C_2_ composite fibers were successfully electrospun from 0 wt% to 10 wt% Ti_3_C_2_ in electrospinning polymer solutions; the morphologies of the electrospun EVOH/Ti_3_C_2_ fibers are exhibited in Figure 1. Between 0 to 10 wt% Ti_3_C_2_, the color of the resulting electrospun fibrous membrane gradually changes from white to gray (Figure 1(a_1_–d_1_)). Specifically, the color of the pristine electrospun EVOH fibrous membrane (0 wt% Ti_3_C_2_) is white, and the color of the electrospun EVOH/Ti_3_C_2_ fibrous membrane is gray. As the Ti_3_C_2_ concentration increases, the composite fibrous membrane gradually changes from light gray to dark gray.

As shown in Figure 1(a_2_–d_2_,a_3_–d_3_), it can be clearly seen that the electrospun EVOH or EVOH/Ti_3_C_2_ fibrous membranes present a three-dimensional non-woven geometry composed of randomly aligned nanofibers. The difference lies in the fact that the pristine electrospun EVOH fibers are smooth and relatively uniform, while the electrospun EVOH/Ti_3_C_2_ fibers are less uniform with fewer Ti_3_C_2_ flakes protruding from the fiber surface. Combined with the high-magnification SEM images, we can see that with the increase in the Ti_3_C_2_ concentration in the electrospinning polymer solution, the Ti_3_C_2_ flakes on the fiber surface also increase. It has been reported that protruding Ti_3_C_2_ flakes can serve as sites for destroying bacterial membranes [34]. However, the presence of the Ti_3_C_2_ flakes results in the inhomogeneity of the fiber (marked by some red dashed circles), which may further affect the mechanical properties of the electrospun EVOH/Ti_3_C_2_ composite fibrous membranes.

Meanwhile, the control of the fiber diameter plays a pivotal role in the specific material properties that show a clear size dependence [40]. The average diameters of the fibers are estimated in Figure 1(a_4_–d_4_). The average fiber diameter decreases as a function of the Ti_3_C_2_ concentration, from 0.82 ± 0.10 μm to 0.60 ± 0.16 μm for the pristine electrospun EVOH fibers and electrospun EVOH/Ti_3_C_2_ composite fibers with 10 wt% Ti_3_C_2_, respectively, suggesting that the addition of the Ti_3_C_2_ results in a decrease in the fiber diameter and an increase in the fiber uniformity. This is due to the addition of Ti_3_C_2_, which increases the conductivity of the polymer solution [30]. The increase in the solution conductivity brings with it a higher amount of carried charge, which allows for the further stretching of the polymer jet, resulting in a smaller fiber diameter [41]. Nevertheless, when the Ti_3_C_2_ increases from 5 wt% to 10 wt%, the average diameter of the fiber decreases only slightly. This phenomenon is due to the fact that when electrospinning a conductive solution, the excess charges in the polymer jet rapidly dissipate to the collector, inducing the collector to produce the opposite charges, resulting in fiber separation, which is not conducive to the formation of fibers [42].

**Figure 2:**
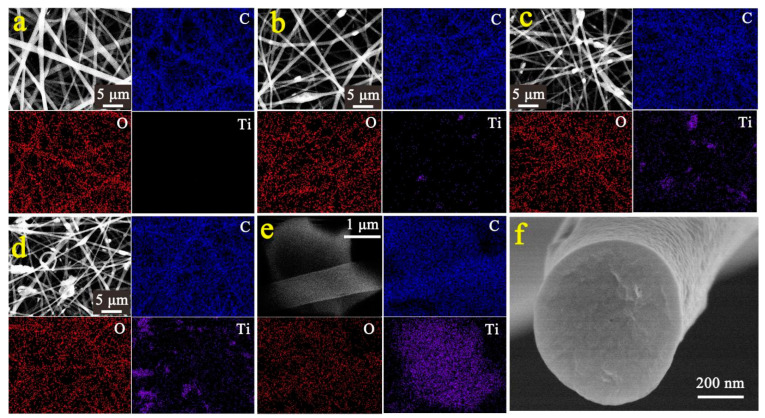
SEM-EDS elemental mapping images of electrospun EVOH/Ti_3_C_2_ membranes exhibiting the distribution of C, O, and Ti in the same area with increasing concentration of Ti_3_C_2_ in the electrospinning solution: (**a**) 0 wt%, (**b**) 2.5 wt%, (**c**) 5 wt%, (**d**) 10 wt%. (**e**) SEM-EDS elemental mapping images, and (**f**) SEM image of electrospun EVOH/Ti_3_C_2_ fiber cross-section with 10 wt% concentration.

In order to confirm the load of the Ti_3_C_2_ in the electrospun EVOH fibers, EDS elemental mapping images were taken, as shown in Figure 2. The pristine electrospun EVOH fibers contain only C and O elements (Figure 1(a_1_–a_4_), while the electrospun EVOH/Ti_3_C_2_ fibers with 2.5 wt% to 10 wt% Ti_3_C_2_ in the electrospinning polymer solution show C, O, and Ti elements (Figure 2b–d), which indicates that Ti element has been loaded onto the fiber. With the increase in the Ti_3_C_2_ concentration, more Ti element is dispersed on the fibrous membrane, especially where the Ti_3_C_2_ flakes are exposed on the fiber surface, Ti element dispersion is more obvious. In addition, an EDS elemental mapping image of the electrospun EVOH/Ti_3_C_2_ composite fiber cross-section with 10 wt% Ti_3_C_2_ is displayed to further detect the Ti_3_C_2_ loading within the fibers. It is confirmed that Ti element is uniformly dispersed in the fiber cross-section and Ti_3_C_2_ flakes are uniformly loaded inside the fibers.

### 3.2. Structure of Electrospun EVOH/Ti_3_C_2_ Composite Fibers

**Figure 3:**
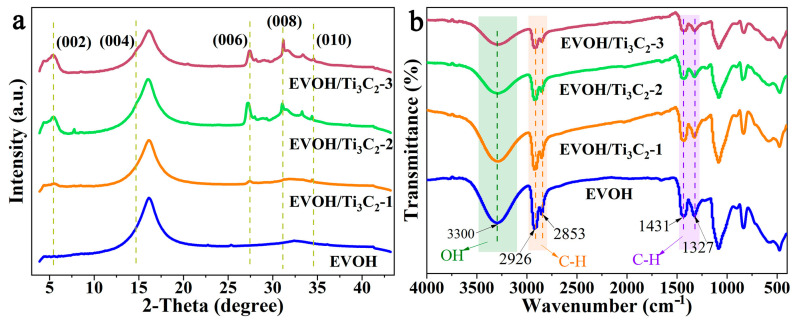
XRD patterns (**a**) and FTIR spectra (**b**) of electrospun EVOH/Ti_3_C_2_ fibers.

To investigate the effect of the Ti_3_C_2_ content on the structure of the electrospun EVOH composite fibers, the XRD patterns and FTIR spectra of the electrospun EVOH/Ti_3_C_2_ fibrous membranes were analyzed, as shown in Figure 3. A typical XRD pattern of the pristine electrospun EVOH fibrous membrane given by Figure 3a displays a crystal peak at a 2θ of about 16.2°, which has been reported in previous literature [21]. With the increase in the Ti_3_C_2_ concentration, the intensity and shape of the diffraction peak appearing at 2θ = 16.2° have no obvious change, indicating that the presence of Ti_3_C_2_ does not affect the crystal structure of the electrospun EVOH fibrous membrane. The 2.5 wt% addition of Ti_3_C_2_ results in the appearance of new crystal peaks, which appear at 2θ = 5.4°, 27.4°, and 34.4°, corresponding to the crystal planes (002), (006), and (010), respectively. As the Ti_3_C_2_ content increases, the intensities of these diffraction peaks increase. At a concentration of 5 wt% Ti_3_C_2_, the new diffraction peak appears at 2θ of 31°, corresponding to the (008) crystal plane. When the concentration of Ti_3_C_2_ increases to 10 wt%, the diffraction peaks at 2θ of 5.4°, 27.4°, 31°, and 34.4° increase, and a new crystal peak appears at 2θ = 14.8°, corresponding to the (004) crystal plane. Previous studies have reported that Ti_3_C_2_ has five diffraction peaks, namely (002), (004), (006), (008), and (010) [29,43]. This manifests that with the increase in the Ti_3_C_2_ concentration, more Ti_3_C_2_ is loaded onto the electrospun EVOH fibrous membrane, and the crystal peaks corresponding to Ti_3_C_2_ are enhanced, meaning that the orientation of Ti_3_C_2_ within the electrospun fiber increases, which has potential applications in the biological field.

The FTIR spectra in Figure 3b show typical characteristic peaks of electrospun EVOH fibrous membrane with vibrational bands at 2926 cm^−1^ and 2853 cm^−1^ for the C-H stretch, 1431 cm^−1^ and 1327 cm^−1^ for bending, which are in agreement with the literature [17,44]. Meanwhile, the basic stretching vibration of the free hydroxyl group (-OH) corresponds to a sharp peak around 3300 cm^−1^ [45]. It can be seen that there are no peaks broadening or shifting with the participation of Ti_3_C_2_, which may be attributed to the low loading of Ti_3_C_2_ in the electrospun EVOH/Ti_3_C_2_ fibers. As a matter of fact, the Ti_3_C_2_ characteristic crystal peaks of the electrospun EVOH/Ti_3_C_2_ fibrous membranes exhibit a lower intensity in their XRD pattern, especially after the addition of 10 wt% Ti_3_C_2_; the corresponding diffraction peaks are not significant (Figure 3a), indicating that although Ti_3_C_2_ has been loaded onto the electrospun EVOH fiber, the low loading in the electrospun EVOH fiber may be the reason for no significant changes in the FTIR. It is worth noting that the typical characteristic peaks of the electrospun EVOH fiber do not change with the increase in the Ti_3_C_2_ concentration, indicating that the addition of Ti_3_C_2_ does not affect the structure of the EVOH fiber.

### 3.3. Thermal Properties of Electrospun EVOH/Ti_3_C_2_ Composite Fibers

**Figure 4:**
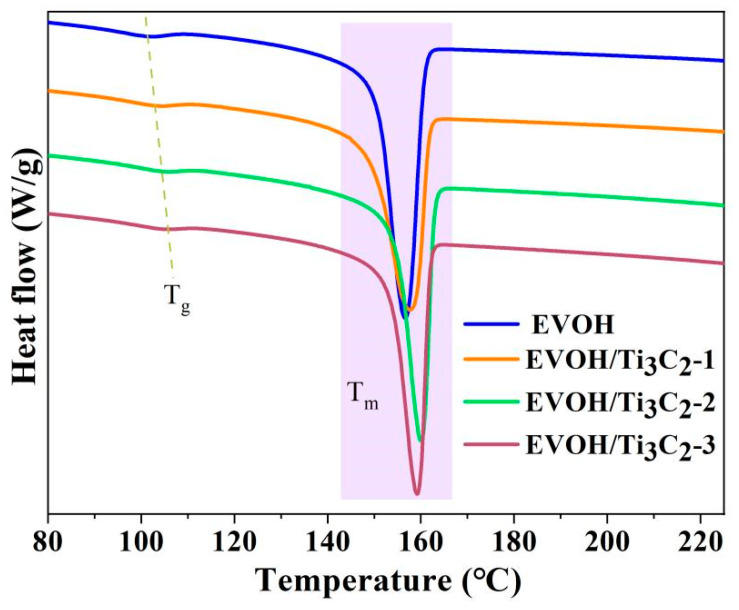
DSC thermograms of electrospun EVOH/Ti_3_C_2_ composite fibers.

DSC analysis was performed to examine the thermal properties of the electrospun EVOH/Ti_3_C_2_ composite fibrous membranes with increasing concentration of Ti_3_C_2_ in the electrospinning solution, as illustrated in Figure 4. As can be seen from the figure that the glass transition temperature and the melting temperature both shift slightly to the right due to the addition of Ti_3_C_2_, that is to say, with the increase in the Ti_3_C_2_ concentration, the glass transition temperature increases, and the melting temperature also increases slightly. In addition, it can be seen from the melting curve shape that the electrospun EVOH/Ti_3_C_2_ composite fibrous membrane with 2.5 wt% concentration of Ti_3_C_2_ can melt more at lower melting temperatures (e.g., 136–149°) in comparison to that of the Ti_3_C_2_ concentration of 5 wt% and 10 wt%, indicating the presence of thin crystals, which is consistent with the XRD results.

The glass transition temperature (T_g_) and melting temperature (T_m_) of the electrospun EVOH/Ti_3_C_2_ fibrous membranes are listed in Table 1. The glass transition temperature and melting temperature of the pristine electrospun EVOH fibrous membrane are 101° and 156°, respectively. With the increasing Ti_3_C_2_ content, the glass transition temperature of the electrospun EVOH/Ti_3_C_2_ fibrous membrane rises to 107°, and the melting temperature slightly increases to 160°. This indicates that the participation of Ti_3_C_2_ increases the thermal stability of the electrospun EVOH fibrous membrane, and the greater the Ti_3_C_2_ concentration in the electrospinning solution, the better the thermal stability of the fibrous membrane.

### 3.4. Wettability of the Fibrous Membranes

The wettability of the electrospun EVOH/Ti_3_C_2_ fibrous membrane were evaluated by the contact angle and fluid uptake ability. By testing the contact angle of the fibrous membrane within 30 min, it is found that the initial contact angle of the pristine electrospun EVOH fibrous membrane is 127°, which decreases to about 124° at 22 min, and then remains unchanged within the 30 min. For the electrospun EVOH/Ti_3_C_2_ fibrous membranes, their initial contact angle is almost the same, at about 125°. The difference is the time when the contact angle of the fibrous membrane changes. The greater the Ti_3_C_2_ content, the faster the contact angle decreases. For example, the electrospun EVOH/Ti_3_C_2_ fibrous membrane with 10 wt% Ti_3_C_2_ in the electrospinning solution exhibits the rapid penetration of the water droplet, that is, the water droplet completely penetrates into the fibrous membrane within 18 min, and the corresponding contact angle is 0°, indicating that the presence of Ti_3_C_2_ improves the hydrophilicity of the electrospun EVOH fibrous membrane.

At the same time, the fluid uptake ability (FUA) of the fibrous membranes was evaluated by immersing the desired fibrous membranes in water to measure their water absorption capacity, according to the following formula [18,19]:

(2) $$FUA(\%)=\frac{(W_{s}-W_{d})\times 100\%}{W_{s}}$$

where W_s_ represents the wet weight of the fibrous membrane, W_d_ represents the dry weight of the fibrous membrane. In this part of the experimental operation, the dry fibrous membrane was first completely immersed in water for 30 s, then removed and placed on absorbent paper for 30 s, and finally weighed, and the above steps were repeated.

**Figure 5:**
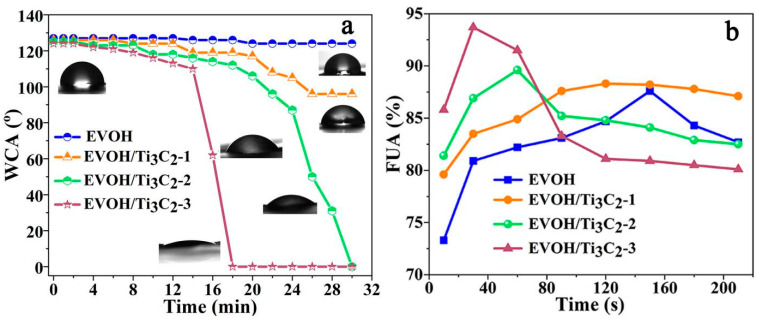
Wettability of electrospun EVOH/Ti_3_C_2_ fibrous membranes: (**a**) the variation and digital images of WCA, (**b**) fluid uptake ability.

As shown in Figure 5b, the pristine electrospun EVOH fibrous membrane achieves absorption saturation in 150 s, with an absorption rate of 87.6%, and then begins to decline. With the increase in the Ti_3_C_2_ concentration, the absorption saturation time of the electrospun EVOH/Ti_3_C_2_ fibrous membrane is shortened, and the corresponding absorption rate is increased. Among them, the absorption saturation time of the EVOH/Ti_3_C_2_ fibrous membrane with 5 wt% Ti_3_C_2_ is half of that of the fibrous membrane with 2.5 wt%, while the electrospun EVOH/Ti_3_C_2_ fibrous membrane with 10 wt% Ti_3_C_2_ reaches absorption saturation in just 30 s, with an absorption rate as high as 93.7%. Based on the contact angle and fluid uptake ability of the fibrous membranes, it can be observed that the wettability of the electrospun EVOH/Ti_3_C_2_ fibrous membrane has been significantly improved due to the introduction of Ti_3_C_2_.

### 3.5. Mechanical Properties of Fibrous Membranes

**Figure 6:**
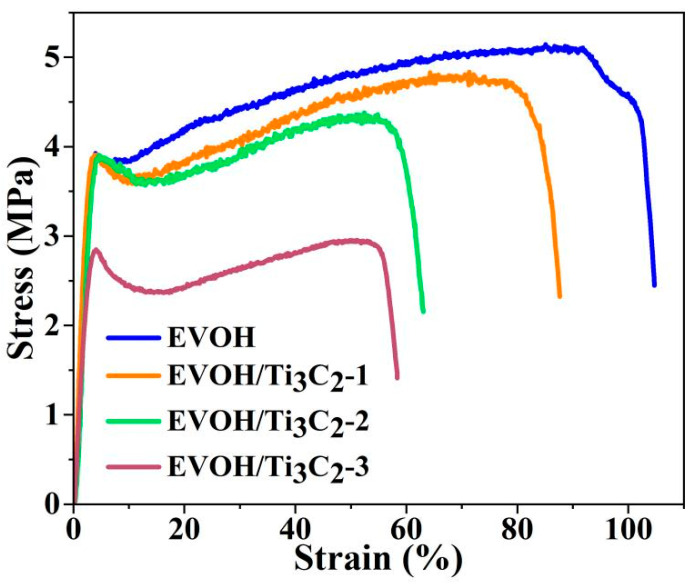
Typical stress–strain curves of electrospun EVOH/Ti_3_C_2_ fibrous membranes.

The mechanical properties of fibrous membranes, which are affected by the morphology and microstructure of fibers, have become an indispensable topic of research for their practical applications [46,47]. Typical stress–stain curves of the electrospun EVOH/Ti_3_C_2_ fibrous membranes were investigated to study the effect of additions of Ti_3_C_2_ on the tensile properties of the electrospun EVOH fibrous membranes, as shown in Figure 6. The tensile curves of the fibrous membranes present some general characteristics, starting with an increase in stress (including a clear linear and a nonlinear elastic stage), then showing a decrease in stress followed by a mild strain hardening [48]. The difference lies in the breaking strength and elongation at the break of the fibrous membrane. It can be seen that the pristine electrospun EVOH fibrous membrane presents a higher tensile stress of 5.13 MPa and a higher breaking elongation of 104%. When the concentration of Ti_3_C_2_ increases from 2.5 wt% to 10 wt%, the tensile stress of the electrospun EVOH/Ti_3_C_2_ fibrous membrane ranges from 4.77 MPa to 2.95 MPa, and the breaking elongation fluctuates from 87.5% to 58.24%, still showing good strength and elasticity. However, compared to the pristine electrospun EVOH fibrous membranes, the tensile stress of the electrospun EVOH/Ti_3_C_2_ fibrous membranes with 2.5 wt% and 5 wt% Ti_3_C_2_ decreased by 7% and 13%, respectively, while the tensile strength of the electrospun EVOH/Ti_3_C_2_ fibrous membranes with 10 wt% Ti_3_C_2_ decreased by 43%, and their corresponding strain also decreased, which indicates that the addition of Ti_3_C_2_ weakens the tensile properties of the fibrous membranes.

Combined with the results in Figure 1, it can be seen that the presence of Ti_3_C_2_ induces the fiber’s thinning, and the smaller the fiber’s diameter, the greater the tensile strength of the corresponding fiber membrane [49,50], which is inconsistent with our tensile results. It is worth noting that although the addition of Ti_3_C_2_ refines the fiber, it causes the fiber to become very uneven. Moreover, the protrusion of some Ti_3_C_2_ flakes on the fiber surface may become the weak points at which the fibrous membrane stretches, and this also weakens the elasticity of the fibrous membrane. Of course, it has also been shown that when the Ti_3_C_2_ concentration is high enough (such as 16 wt%), the Ti_3_C_2_ flakes are interconnected along the fiber axis, which will increase the uniformity of the fibrous membrane and may improve the strength of the fibrous membrane [29].

### 3.6. Air Permeability and Water Vapor Permeability of Fibrous Membrane

**Figure 7:**
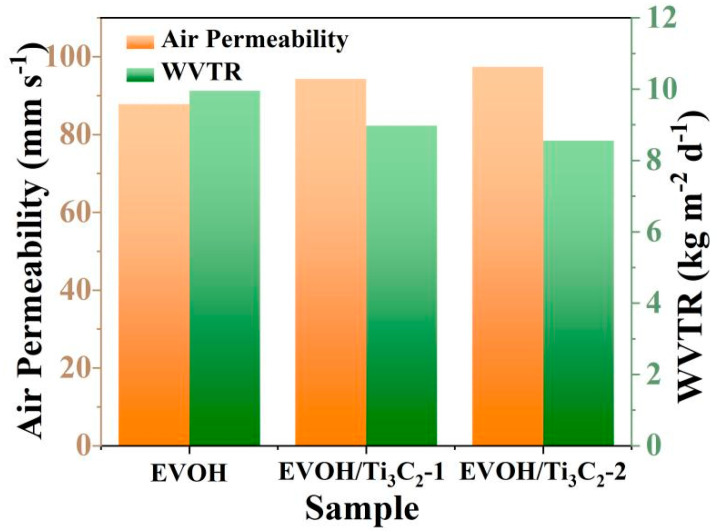
Air permeability and WVTR of electrospun EVOH/Ti_3_C_2_ fibrous membranes.

The air permeability and water vapor permeability of the electrospun EVOH/Ti_3_C_2_ fibrous membranes were further estimated. As can be seen from Figure 7, the electrospun EVOH/Ti_3_C_2_ fibrous membranes display excellent air permeability and water vapor permeability. When the Ti_3_C_2_ concentration increases from 0 wt% to 5 wt%, the air permeability of the fibrous membrane elevates from 87.8 mm s^−1^ to 97.4 mm s^−1^, while the WVTR of these membranes declines from 10 kg m^−2^ d^−1^ to 8.6 kg m^−2^ d^−1^, implying that the addition of Ti_3_C_2_ improves the air permeability of the electrospun EVOH fibrous membranes. It has been reported that the air permeability and water vapor permeability of the electrospun fibrous membranes are linearly positive related to the porosity, that is, the more pores in the fibrous membranes, the more air molecules and water vapor will pass through [51]. Figure 5 shows that Ti_3_C_2_ can rapidly improve the hydrophilic properties of the electrospun EVOH fibrous membrane, evidencing that it is a hydrophilic material. When the fibrous membranes are tested for water vapor permeability, the Ti_3_C_2_ flakes will absorb some water vapor, which may result in less water vapor passing through the fibrous membranes.

## 4. Conclusions

In summary, we have successfully prepared an EVOH/Ti_3_C_2_ composite fiber for the first time via a one-step electrospinning method and further investigated the effect of different Ti_3_C_2_ concentrations on the structure and properties of the electrospun EVOH/Ti_3_C_2_ fibrous membranes. The added Ti_3_C_2_ was well loaded into the fibers and induced a reduction in the fiber diameter from 0.82 μm to 0.60 μm. Moreover, Ti_3_C_2_ could self-orient along the fiber axis but did not change the crystalline structure of the electrospun EVOH fiber, improving the crystallinity and thermal stability of the composite fibers. As the concentration of Ti_3_C_2_ increased, the contact angles of the electrospun EVOH/Ti_3_C_2_ fibrous membranes decreased from 125° to 0° within 18 min, and these fibrous membranes reached water absorption saturation within 30 s, with a UFA as high as 93.7%, demonstrating the excellent wettability of electrospun EVOH/Ti_3_C_2_ fibrous membranes. Meanwhile, the electrospun EVOH/Ti_3_C_2_ fibrous membranes also showed outstanding air permeability and water vapor permeability, as well as modest mechanical properties. The as-prepared electrospun EVOH/Ti_3_C_2_ fibrous membranes could serve as a multi-functional textile for the potential candidates in bioprotective materials, dressing materials, etc., laying the foundation for the further development of applications in later work.

## Figures and Tables

**Table 1:** DSC results (i.e., T_g_ and T_m_) of electrospun EVOH/Ti_3_C_2_ fibrous membranes.

Samples	T_g_ (°C)	T_m_ (°C)
EVOH	101	156
EVOH/Ti_3_C_2_-1	104	158
EVOH/Ti_3_C_2_-2	105	160
EVOH/Ti_3_C_2_-3	107	159

## Data Availability

Data are contained within the article.
